# An Obstetrical Work from the Government Printing Office

**Published:** 1873-12-01

**Authors:** 


### Medical Examiner.

*A Semi-Monthly Journal of Medical Sciences.*

EDITED BY

N. S. DAVIS, M. D., AND F. H. DAVIS, M. D.

Chicago, December 1st, 1873.

### EDITORIAL.

### AN OBSTETRICAL WORK FROM THE GOVERNMENT PRINTING OFFICE.

Some weeks ago we received from the Government Printing Office a folio volume of about five hundred pages, entitled "Report of the Columbia Hospital for Women, and Lying-in-Asylum, Washington, D. C. By J. Henry Thompson, Surgeon-in-Chief."

We refrained from saying anything, either *pro* or *con*, regarding the merits of the work. Some of the journals, however, have been less reserved in expressing their opinions, and, altogether, the work has received a pretty severe handling from the medical press of the country. *The Clinic*, of Cincinnati, quotes from a German paper of that city some comments regarding the book, of which the following are specimens:

"Why, among all the physicians and accoucheurs of this country, just this Thompson in Washington was chosen to have his cases printed at the cost of the Government, and with edifying illustrations, we do not know. We do not believe that children come into the world in Washington differently than in less favored parts of the country. The ways of our Administration are mysterious; and as it has already stretched out its hand after the telegraph and savings-bank business; as it wishes to build canals and railroads, and strives to regulate the money and gold market; as it "protects" manufacturers, and creates National banks; as it ought to irrigate sandy deserts, and in general do everything for everybody, it is a matter of course that it should receive human beings on their birth, and should seek to provide that they come into the world in the correct way.

"Perhaps, merely because our intellect is too limited, we are not able to comprehend the blessings of such a system. We are of opinion that medical works have hitherto been printed by private means, and have found the necessary sale; and that this can remain so for the future. We are further of the opinion that every physician can buy the works he needs himself, and need not obtain them at the cost of the tax-payer. We are further of the opinion that the Government cannot possibly present such a book to every doctor in the United States, but that it would be extremely unjust to present anything to one which it is not ready to give to another. In respect to the work in question, it cannot be asserted that it contains especially important or valuable facts. One of the professors in a medical college here assures us that it contains by no means extraordinary cases, about which one could not learn what is necessary from other sources. The majority of our physicians are of the opinion that it is only a puff for Dr. Thompson, who has the fortune to belong to the favorites of the Administration.

"The printing of such works at the expense of the Government is nothing else than a part of the system of extravagance, preference, and wastefulness under which the country suffers. The Government printing press squanders yearly millions of dollars for books and documents which usually are not worth a hundredth part of what they cost."

